# Successful management of hamstring injuries in Australian Rules footballers: two case reports

**DOI:** 10.1186/1746-1340-13-4

**Published:** 2005-04-12

**Authors:** Wayne T Hoskins, Henry P Pollard

**Affiliations:** 1 Macquarie Injury Management Group, Department of Health & Chiropractic, Macquarie University, NSW 2109, Australia

**Keywords:** hamstring, treatment, sports injury, chiropractic, manipulation, football

## Abstract

Hamstring injuries are the most prevalent injury in Australian Rules football. There is a lack of evidence based literature on the treatment, prevention and management of hamstring injuries, although it is agreed that the etiology is complicated and multi-factorial. We present two cases of hamstring injury that had full resolution after spinal manipulation and correction of lumbar-pelvic biomechanics. There was no recurrence through preventative treatment over a twelve and sixteen week period. The use of spinal manipulation for treatment or prevention of hamstring injury has not been documented in sports medicine literature and should be further investigated in prospective randomized controlled trials.

## Introduction

Hamstring injuries are the most prevalent injury in Australian Rules football [1,2]. This may be possibly due to the unique physical demands of the game requiring rapid acceleration, endurance and agility running, kicking and bending to pick up the ball. Hamstring injuries are not confined strictly to Australian Rules football but are also seen in soccer [3], athletics [4], hurling [5], cricket [6] and touch football [7]. This makes hamstring injuries the most prevalent muscle injury in sports consisting of rapid acceleration and maximum speed running. Such injuries can and do result in significant financial consequences to players and clubs alike.

It is agreed that hamstring injuries have a complicated multi-factorial etiology, including muscle weakness and balance, lack of warm up, decreased flexibility, previous injury history and fatigue [8]. The only conclusive risk factors for future injury is a current hamstring injury or a previous history of hamstring injury [1,9]. This makes prevention of the initial injury a primary focus in management efforts. The purpose of this paper is to present two cases of hamstring injury that were effectively managed with spinal manipulative therapy (SMT) and correction of lumbar-pelvic biomechanics. Prevention of re-injury may have been due to ongoing maintenance type care.

### Back related hamstring injury

Some authors have listed a separate category of hamstring injury known as a 'back related hamstring injury' which is classified as having both local hamstring signs and positive lumbar signs [9,10]. It is known that referred myotomal pain from lumbar-pelvic structures, the sciatic nerve and the gluteal or piriformis muscles can mimic hamstring strains [9]. The world's longest serving injury surveillance, performed by the elite Australian Football League (AFL) uses an umbrella term for hamstring injury which fails to differentiate the potential diagnoses. This means the true prevalence of back-related hamstring injuries in Australian Rules footballers is unknown. Using MRI to confirm the diagnosis of hamstring injury, 19% are without muscle damage [3], suggesting no local muscle pathology and injury to be related to altered functional biomechanics or pain referral that does not appear on cross sectional imaging. This type of injury would logically require different forms of treatment than simple muscular-tendon injuries. It has been postulated that hamstring injuries may have a biomechanical basis and therefore it is reasonable to suggest that assessment of hamstring injury should include a biomechanical evaluation, especially that of the lumbar spine, pelvis and sacrum [3].

There is a paucity of literature about the role of aberrant lumbar-pelvic biomechanics as an etiological factor predisposing to hamstring injury. It is tempting to speculate that this may explain why hamstring injuries have the highest recurrence rate of any injury in the AFL. Thirty three per cent of injured players are likely to re-injure their hamstring on return to competition and miss subsequent matches [1]. A significant risk of injury recurrence exists in the first few weeks following return to play, with the cumulative risk of recurrence for the remainder of the season being 30.6% [11]. No significant change in recurrence rates has been noted over the last 7 years, while players are missing more time on average due to injury [1,12]. In contrast, other injuries in the AFL have noted a considerable improvement in decreased rates of recurrence over this time frame [12]. This suggests that players are being managed more conservatively with regards to return to competition from hamstring injuries and there appears to be no change in the treatment protocol if recurrence rates have yet to decline. This may suggest the possibility of a biomechanical factor that may require a differing approach that has yet to be introduced. No prevention effort will be successful without understanding the etiological factors predisposing hamstring injury and efforts to decrease recurrence rates for hamstring injuries will be unsuccessful if the possibility of a biomechanical factor is excluded in the etiology.

### Case Report 1

A 19-year-old male, semi-elite Australian Rules footballer presented with left sided hamstring pain that occurred during a game two weeks prior. The patient had not played a game or been able to train for two weeks since the injury. He had been treated with cryotherapy, NSAID's, slump stretching, lumbar spine mobilizations, ultrasound and massage to the hamstrings. He had a history of mild osteitis pubis 12 months previously that was treated with rest and modified activity. There had been no prior history of hamstring or low back injury.

On physical examination the patient was standing with an apparent lumbar spine hyperlordosis, anterior pelvis tilt, flexed knees and increased thoracic kyphosis. There was tight (reduced range of motion) bilateral hip flexors (modified Thomas position* +15°) and hamstring muscles (45° straight leg raise [SLR]), hypertonicity of the gluteii, hamstring, lumbar and psoas muscles and general mid thoracic and lumbar spine motion restriction, determined by inter-segmental motion palpation and observation of range of motion (ROM). (*Modified Thomas testing requires the patient to sit at the edge of the table and to bring one knee to their chest to firmly flatten their back. They then assume the supine position, allowing the testing leg to extend off the table. An angle is formed between the femur and a line drawn parallel to the tabletop. A positive angle means the femur is projecting upwards. A negative angle means the femur hangs downwards). There was weakness of the left hamstring and gluteus maximus graded 4/5. Hamstring tenderness could not be localized on palpation. Other physical examination findings, including Trendelenburg, valsalva, neurological, slump, extension leg raise and hip and sacroiliac joint motion palpation and orthopedic testing were unremarkable. The patient was given a working diagnosis of back-related hamstring injury as a result of lumbar-pelvic myofascial pain referral, mimicking a grade one hamstring strain. Differential diagnoses included pain referral from gluteal trigger points.

Treatment involved long-lever SMT to the lumbar spine, short-lever SMT to the mid thoracic spine, drop piece knee manipulation, active release soft tissue massage techniques (ART) to the psoas, gluteal, lumbar and hamstring muscles and proprioceptive neuromuscular facilitation (PNF) stretching of the hamstring and psoas muscles. Post treatment, modified Thomas position bilaterally was +5°, SLR 60° bilaterally and muscle strength was graded 5/5.

The patient received 3 treatment sessions that week and played a match the next week without re-injury. He was put on a maintenance program for the rest of the 12 weeks of the season including finals (one visit per week for a month, one visit per fortnight thereafter) which included the above treatment and strengthening and muscle activation work (to improve hip extension and abduction motor patterns) to the gluteus maximus and medius, multifidus, transversus abdominus and internal oblique muscles. Maximum medical improvement (MMI) was reached after 7 treatments. The patient finished the season without re-injury. Posture and muscle length changes continued to improve over this period (bilateral modified Thomas position -5°, SLR 85°).

### Case Report 2

A 25 year old male, semi-elite Australian Rules footballer felt a 'twinge' in his right hamstring during a game. He presented to us the day after, complaining of tightness in his medial right hamstring and a stiff low back. He had no previous history of hamstring injury but had suffered episodic low back pain over a 5-year period.

On physical examination, the right pelvis was low compared to the left in standing position; there were tight right (45° SLR) and left (55° SLR) hamstrings and tight left hip flexors (+10° modified Thomas position). There was palpable hypertonicity through the right hamstring, left psoas, lumbar and gluteal muscles, thoracolumbar spine and right sacro-iliac joint (SIJ) motion restriction, positive Gillett's (standing S-I joint motion palpation) and extension leg raise testing for the right SIJ and weakness of the right hamstring and gluteus maximus muscles rated 4/5. Hamstring tenderness could not be localized on palpation but mild discomfort was reproduced in resisted muscle testing. Other physical examination findings and testing procedures, including Trendelenburg, valsalva, neurological, slump, lumbar ROM and hip joint motion palpation and orthopedic testing were unremarkable. The patient was diagnosed with a back-related hamstring injury on the basis of his apparent right SIJ motion restriction and pain referral. Differential diagnoses included pain referral from lumbar-pelvic myofascial structures, gluteal trigger points or a grade 1 hamstring injury with concurrent lumbar-pelvic dysfunction.

Treatment involved high velocity low amplitude (HVLA) SMT to the right SIJ and thoracolumbar spine, long axis manipulation to the right hip joint, ART to the right hamstring, left psoas, lumbar and gluteal muscles, PNF stretching of the right hamstring and left psoas and hamstring cryotherapy. Post treatment, modified Thomas position was 0° on the left, SLR 55° on the right and 65° on the left. Muscle strength was graded 5/5.

The patient did not participate in training during the week and received 2 more treatment sessions. He played a match the next weekend without re-injury. He was seen twice the next week and put on a maintenance program for the 16 weeks remaining in the season (one visit per week for a month, one visit per fortnight for a month, one visit per month thereafter). This included the above treatment plus strengthening and muscle activation work (to improve hip extension motor patterns and running technique) to the gluteus maximus, multifidus, transversus abdominus and internal oblique muscles and home advice including flexibility and stability work. MMI was reached after 10 treatments. No re-injury occurred during this period and muscle length changes continued to improve (bilateral modified Thomas position 0°, SLR 75°).

## Discussion

In the sports medicine literature, spinal manipulation for the treatment or prevention of hamstring injury has not been documented, despite it being frequently used by chiropractors and other manual therapists. In fact, there is a lack of literature on the management of hamstring injuries in general. A recent review of the literature suggested that low back pain from the zygopophyseal joints at the levels of spinal nerve roots supplying the hamstrings may provoke local muscular responses such as increased muscle tension which may predispose injury [8]. However, this potential association with injury is yet to be scientifically validated. The only treatment methods that have been documented in randomized controlled trials are slump stretching [13,14] and rehabilitation protocols [15]. Slump stretching involves maximal cervical, thoracic and lumbar flexion with full hip flexion, knee extension and ankle dorsiflexion with passive practitioner overpressure. These studies have had low subject numbers, making conclusions weak.

The slump test is said to significantly differentiate referred posterior thigh pain from that due to muscular-tendon strain [13] and has been able to identify those with recurrent hamstring strains in a small study [14]. Slump stretching as a treatment procedure (when slump testing is positive) has been shown to be more beneficial in returning athletes to competition than standard physiotherapy treatment alone (ultrasound, massage, progressive flexibility and strengthening) [13]. The slump test has been proposed to be a measure of 'neural tension' which is postulated to predispose hamstring injury [14]. However, the anatomical relationship of the hamstrings with the thoracolumbar fascia (TLF) system has been neglected. The tendon of bicep femoris is continuous with the sacrotuberous ligament, passing across the sacrum and attaching to the thoracolumbar TLF [16]. This functionally connects the hamstrings to the lumbar spine, upper torso, shoulder and occiput and casts doubt on reliability of the slump test as being able to measure neural tension [17]. Contracture of the muscular attachments of the TLF has been documented to cause TLF its displacement [16]. Therefore neural tension may only be an assumption and it may more likely be myo-fascial tension, or possibly a combination of the two giving a positive slump test. Postural changes such as forward weight bearing, as occurs during forward lean gait, will also cause hamstring tension and predispose hamstring injury. This suggests that the TLF system should be assessed during treatment of hamstring injuries.

Australian Rules footballers with a previous back injury have been found to have a significant increased risk of hamstring injury [9]. A strong relationship between age and hamstring, calf and Achilles injuries (with a L5 and S1 nerve supply) also exists in AFL players [18]. The L4/5 and L5/S1 levels are the most common areas for spinal degeneration and athletes are susceptible to degenerative changes at an earlier age than the normal population [19]. Altered neural input from the levels that innervate hamstrings may be causing and prolonging hamstring injuries. Long term prospective studies are required to further investigate this finding.

Significant excessive lumbar lordosis has been found retrospectively in athletes with previous hamstring injury when compared to a group with no injury history [20]. Prospectively, thigh injuries as a group (hamstring, quadriceps and adductor injuries) have been linked to postural defects, including increased lordosis, sway back and knee interspace measurements [21], while the incidence of muscle injuries in general has been linked to the existence of defective body mechanics associated with the site of injury [22]. This indirectly suggests that improving lumbar-pelvic biomechanics may play a role in treatment and prevention of hamstring injury.

Of the other risk factors linked with hamstring injuries, low hamstring strength is a risk factor with some degree of clinical evidence [23]. Strength deficits have been found to exist in athletes with a history of recurrent hamstring strain [24]. This may have been the cause of the initial injury, be due to weakness from ineffective rehabilitation or from dysfunction in the lumbar spine, SIJ or pelvis. An association between altered pelvic function and hamstring injury is suggested by a past history of groin injury and osteitis pubis being significant risk factors for hamstring injury [9]. Although it is only an assumption that pelvic problems contribute to groin injuries through its kinematic chain relationship. One small randomized clinical trial has looked at the effectiveness of manipulation targeted at the SIJ for the treatment of hamstring injuries [25]. The manipulation group improved hamstring strength compared to the control group, suggesting SIJ dysfunction may be related to initial hamstring injury.

We believe that the two cases we saw and treated were related to a lumbar-pelvic biomechanical aspect. In our two cases, there existed clinically either lumbar hyperlordosis, anterior pelvis tilt or lateral pelvis tilt. This is consistent with the findings of Hennessy and Watson (1993) and Watson (1995, 2001). Improvement of these biomechanical factors, including the use of SMT, resulted in successful treatment and prevention of the hamstring injuries. This leads us to hypothesize that inter-segmental and/or global lumbar-pelvic biomechanical dysfunction produced either referred pain or hamstring muscle insufficiency via the TLF as a cause of the hamstring injuries and possibly why these cases did not improve with previously used standard treatment modalities. There are limitations to this hypothesis including the reliability (or lack thereof) of the diagnosis of mechanical dysfunction of the low back and pelvic areas. To conclude that there is mechanical dysfunction in the low back particularly in the absence of pain also needs further research.

## Conclusion

Hamstring injuries have a complex multi-factorial etiology. Two forms of hamstring injury have been identified with potentially different pathogenesis, notionally requiring different treatment methods. From our case reports and evidence presented, it appears that spinal manipulation and improving lumbar-pelvic biomechanics and function may play a role in treatment and prevention of hamstring injury. This should be further investigated in prospective, randomly controlled trials with long-term follow up. Given that a recurrence rate exists for hamstring injuries, the possibility that a concomitant biomechanical aspect exists should be pursued.

## References

[B1] Orchard J, Seward H (2002). Epidemiology of injuries in the Australian Football League, seasons 1997–2000. Br J Sports Med.

[B2] Hoskins W, Pollard H (2003). Injuries in Australian Rules football: A review of the literature. Aust Chiro Osteo.

[B3] Woods C, Hawkins RD, Maltby S, Hulse M, Thomas A, Hodson A (2004). The Football Association Medical Research Programme: an audit of injuries in professional football – analysis of hamstring injuries. Br J Sports Med.

[B4] McLennan JG, McLennan JE (1990). Injury patterns in Scottish heavy athletics. Am J Sports Med.

[B5] Watson AW (1996). Sports injuries in school gaelic football: a study over one season. Ir J Med Sci.

[B6] Stretch RA (2003). Cricket injuries: a longitudinal study of the nature of injuries to South African cricketers. Br J Sports Med.

[B7] Neumann DC, McCurdie IM, Wade AJ (1998). A survey of injuries sustained in the game of touch. J Sci Med Sport.

[B8] Croisier JL (2004). Factors associated with recurrent hamstring injuries. Sports Med.

[B9] Verrall GM, Slavotinek JP, Barnes PG, Fon GT, Spriggins AJ (2001). Clinical risk factors for hamstring muscle strain injury: a prospective study with correlation of injury by magnetic resonance imaging. Br J Sports Med.

[B10] Orchard JW (2001). Intrinsic and extrinsic risk factors for muscle strains in Australian football. Am J Sports Med.

[B11] Orchard J, Best TM (2002). The management of muscle strain injuries: an early return versus the risk of recurrence. Clin J Sports Med.

[B12] Orchard JW, Seward H (2003). AFLMOA. AFL Injury Report.

[B13] Kornberg C, Lew P (1989). The effect of stretching neural structures on grade one hamstring strains. J Orthop Sports Phys Ther.

[B14] Turl SE, George KP (1998). Adverse neural tension: a factor in repetitive hamstring strain?. J Orthop Sports Phys Ther.

[B15] Sherry MA, Best TM (2004). A comparison of 2 rehabilitation programs in the treatment of acute hamstring strains. J Orthop Sports Phys Ther.

[B16] Vleeming A, Pool-Goudzwaard AL, Stoeckart R, van Wingerden JP, Snijders CJ (1995). The posterior layer of the thoracolumbar fascia. Its function in load transfer from spine to legs. Spine.

[B17] Barker PJ, Briggs CA (1999). Attachments of the posterior layer of lumbar fascia. Spine.

[B18] Orchard JW, Seward H (2002). AFLMOA. AFL Injury Report.

[B19] Ong A, Anderson J, Roche J (2003). A pilot study of the prevalence of lumbar disc degeneration in elite athletes with lower back pain at the Sydney 2000 Olympic Games. Br J Sports Med.

[B20] Hennessey L, Watson AW (1993). Flexibility and posture assessment in relation to hamstring injury. Br J Sports Med.

[B21] Watson AW (1995). Sports injuries in footballers related to defects of posture and body mechanics. J Sports Med Phys Fitness.

[B22] Watson AW (2001). Sports injuries related to flexibility, posture, acceleration, clinical defects, and previous injury, in high-level players of body contact sports. Int J Sports Med.

[B23] Orchard J, Marsden J, Lord S, Garlick D (1997). Preseason hamstring muscle weakness associated with hamstring muscle injury in Australian footballers. Am J Sports Med.

[B24] Croisier JL, Forthomme B, Namurois MH, Vanderthommen M, Crielaard JM (2002). Hamstring muscle strain recurrence and strength performance disorders. Am J Sports Med.

[B25] Cibulka MT, Rose SJ, Delitto A, Sinacore DR (1986). Hamstring muscle strain treated by mobilizing the sacroiliac joint. Phys Ther.

